# Insight Into Whole Genome of *Aeromonas veronii* Isolated From Freshwater Fish by Resistome Analysis Reveal Extensively Antibiotic Resistant Traits

**DOI:** 10.3389/fmicb.2021.733668

**Published:** 2021-09-17

**Authors:** Rungnapa Sakulworakan, Putita Chokmangmeepisarn, Nguyen Dinh-Hung, Elayaraja Sivaramasamy, Ikuo Hirono, Rungthip Chuanchuen, Pattanapon Kayansamruaj, Channarong Rodkhum

**Affiliations:** ^1^The International Graduate Program of Veterinary Science and Technology (VST), Faculty of Veterinary Science, Chulalongkorn University, Bangkok, Thailand; ^2^Center of Excellent in Fish Infectious Diseases (CE FID), Department of Veterinary Microbiology, Faculty of Veterinary Science, Chulalongkorn University, Bangkok, Thailand; ^3^Tokyo University of Marine Science and Technology, Minato-ku, Japan; ^4^Department of Veterinary Public Health, Faculty of Veterinary Science, Chulalongkorn University, Bangkok, Thailand; ^5^Department of Aquaculture, Faculty of Fisheries, Kasetsart University, Bangkok, Thailand

**Keywords:** *Aeromonas veronii*, antimicrobial resistance, genomic islands, resistome analysis, tilapia, whole genome sequencing

## Abstract

*Aeromonas veronii* outbreaks in tilapia farming caused relatively high mortalities, and the bacteria was resistant to many kinds of antimicrobials used in Thailand aquaculture. According to the CLSI standard, the determination of antimicrobials efficacy has been limited to phenotypic analyses, and a genomics study is required. This research aimed to analyze the resistome of *A. veronii* isolated from diseased tilapia in Chainat, Nong Khai, and Uttaradit provinces in Thailand. A total of 12 isolates of *A. veronii* were identified based on the *gyrB* sequencing and then, the MIC values to eight antimicrobials (AMP, AML, GEN, ENR, OXO, OTC, SXT, and FFC) were determined. According to the MIC patterns, whole genome sequencing (WGS) of five representatives and resistome analysis were performed, including 15 genomes of *A. veronii* isolated from freshwater fish available in the NCBI. All tilapia isolates were susceptible to FFC but resistant to AML and AMP while OTC resistance was the most dominant. In addition to the WGS analysis, 4.5 Mbp of *A. veronii* was characterized. A total of 20 ARGs were detected by resistome analysis and 16 genes were shared among the *A. veronii* population. In conclusion, *A. veronii* strains isolated from tilapia exhibited a resistance to several antimicrobials and multidrug resistance (MDR) which was related to the presence of multiple ARGs. *Aeromonas veronii* shared the ARGs in their population worldwide with a possibility of a plasmid-mediated acquisition due to the presence of resistance islands.

## Introduction

Thailand is an important aquaculture producer and one of the top world fish exporting countries (Food and Agriculture Organization (FAO), 2018). The total production of Nile tilapia (*Oreochromis niloticus*) reached 217.9 metric tons in 2017 and was regarded the highest among other economically important freshwater aquaculture (52.7% of the total production yield) (Department of Fisheries (DOF), 2019). Nowadays, inland intensive farming system has become the standard practice for Nile tilapia production which corresponds to the continual increase of consumer demand. However, the intensive farming can lead to more stressful conditions and eventually bring the risk of infectious diseases which threatens the tilapia industry as a whole (Dong et al., 2015). *Aeromonas veronii*, an etiological organism of Motile Aeromonas Septicemia (MAS), contributed to major losses all-year-round with the cumulative mortality varying from 10 to 100% (Dong et al., 2017). More importantly, these bacteria can also cause disease in a wide range of hosts such as human, amphibian, and other aquatic animals (Martino et al., 2016). Antimicrobial therapy is a standard practice for controlling bacterial diseases occurring in tilapia farms. However, several antimicrobial groups that are allowed for use in Thailand aquaculture have also been widely used in human medication such as Beta-lactam, Quinolone, Tetracycline, or Sulfonamides (FCSTD, 2012). The situation of antimicrobial resistance (AMR) and multi drug resistance (MDR; resistant to at least one drug in three or more group of antimicrobials) (Magiorakos et al., 2012) has not only been found in human medicine but also in aquaculture as well, with the report of resistant isolates from different countries (Caruso, 2016; Deng et al., 2016; Baron et al., 2017; Yang et al., 2017; Roh et al., 2019). Wastewater from aquaculture may contain drug residues and resistant bacteria which subsequently pass on to the environment and, ultimately, the consumers. The AMR property can be transferred horizontally to other terrestrial bacteria through the mobile genetic elements (MGEs) (Subramani and Michael, 2017). *Aeromonas* spp. has been well-known as potential bacteria harboring several AMR determinants including plasmid, integron, and genomic island (Piotrowska and Popowska, 2015). However, concerning the current situation of *A. veronii* outbreaks in Thailand, antimicrobial susceptibility is very limited with little available AMR information. Therefore, this research aimed to gain the comprehensive understanding of AMR, as well as the potentially correlated genetic determinants in the recent isolates of *A. veronii* isolated from tilapia using a genome-wide investigation of the AMR genetic determinants (resistome analysis). Due to the potency of whole genome sequencing, WGS can simultaneously investigate the collection of resistance-associated genes which can lead to the *in silico* prediction of AMR traits (Zankari et al., 2012). Nevertheless, the information of intrinsic and acquired resistance genes can be beneficial to the public health concern by revealing the potential transferable AMR determinants in *A. veronii* and increases the awareness of the possible distribution of AMR traits in the future.

## Materials and Methods

### Bacterial Isolates

A total of 12 isolates of *Aeromonas veronii* were obtained from the bacterial collection of FID-RU (Fish Infectious Disease Research Unit, Chulalongkorn University, Thailand), which were isolated from Nile tilapia (*Oreochromis niloticus*) and hybrid red tilapia (*O. mossambicus* × *O. niloticus*) during the disease outbreaks from 2015 and 2018 in Thailand. The details of each isolate are presented in Table 1. The bacterial isolation method and biosecurity were approved by Chulalongkorn University, Faculty of Veterinary Science Institutional Biosafety Committee (CU-VET-IBC) under the permit ID: IBC 183104.

**TABLE 1:** Details of *Aeromonas veronii* isolates used in this study.

**Year**	**Location**	**Host**	**Clinical manifestation**	**Chemical usage**	**Isolate**	**Reference**
2015	Nong Khai, Thailand	Nile tilapia	NA	NA	NK01	Dong et al., 2015
					NK02	
					NK03	
					NK04	
					NK05	
					NK06	
					NK07	
2018	Uttaradit, Thailand	Hybrid red tilapia	Hemorrhage on skin kidney and fin	OTC and Vitamin C	UDRT09	This study
2018	Chainat, Thailand	Hybrid red tilapia	Hemorrhage on skin and kidney enlargement with fin rot	OTC	CNRT07	This study
					CNRT11	
					CNRT12	
					CNRT13	

*NA, not applicable; OTC, oxytetracycline.*

### Taxonomic Identification

*Aeromonas veronii* stocks (TSB with 20% of glycerol) were recovered by streaking on a tryptic soy agar (TSA; Difco, United States) with an overnight incubation at 28°C. A single colony was then inoculated into 3 mL of tryptic soy broth (TSB; Bacto, United States) and incubated at 28°C for 24 h with shaking at 160 rpm. Bacterial suspension was used for DNA extraction using the Wizard Genomic DNA purification kit (Promega Corporation, United States) following the instructions of the manufacturer. To confirm the bacterial taxonomy, partial *gyrB* (∼1.4-kb-long) was amplified using the universal primers (*gyrB*3F: TCC GGC GGT CTG CAC GGC GT and *gyrB*14R: TTG TCC GGG TTG TAC TCG TC) with PCR conditions described in the original publication (Hoel et al., 2017). PCR product was separated on 1% agarose Tris-borate-EDTA gel using NucleoSpin^®^ Gel and PCR cleanup (Macherey-Nagel, United States). The purified amplicon was submitted to FirstBASE laboratory service (Malaysia) for Sanger sequencing. The reads were assembled into a contiguous sequence using the ContigExpress software. The identity of the contig was searched against the NCBI nucleotide database using Megablast. Species level demarcation cut-off was set to a sequence coverage of ≥99% (Hoel et al., 2017).

### Determination of the Minimum Inhibitory Concentration

A total of 12 isolates from the glycerol stock were recovered on the TSA supplemented with 5% sheep blood and incubated at 28°C for 24 h (Hassan et al., 2017). Each single colony was sub-cultured on the Mueller-Hinton agar (MHA) for the MIC determination by broth microdilution assay with eight antimicrobials including Amoxicillin, AMC [256–0.5 mg/L]; Ampicillin, AMP [256–0.5 mg/L]; Enrofloxacin, ENR [16–0.03 mg/L]; Florfenicol, FFC [64–0.125 mg/L]; Gentamicin, GEN [256–0.5 mg/L]; Oxolinic acid, OXO [64–0.125 mg/L]; Oxytetracycline, OTC [256–0.5 mg/L]; and Sulfamethoxazole + trimethoprim, SXT [256–0.5 mg/L] (Oxoid, United Kingdom) according to the CLSI guideline VET04 (Clinical and Laboratory Standards Institute (CLSI), 2014). The concentration of the bacterial suspension was adjusted by aliquoting 0.85% normal saline to obtain 0.5 McFarland turbidity. Subsequently, the adjusted bacterial suspension was mixed with the cation-adjusted Mueller-Hinton broth (CAMHB) containing an antimicrobial agent at a ratio of 1:1 (V/V) (Baron et al., 2017). Incubation was carried out at 28°C for 24 h. The MIC value was interpreted from a visible growth of bacteria in the medium solution. The resistance trait of each bacterial isolate was categorized into either resistant, sensitive, or multidrug resistant (at least one drug from three or more antimicrobial class) based on the epidemiological cut-off value of the *Aeromonas* species published previously (Baron et al., 2017). Herein, *Escherichia coli* ATCC 25922 was used as an internal control of this MIC assay.

### Whole Genome Sequencing

Five representative *A. veronii* isolates, namely NK01 (resistant to three antimicrobial classes, 3MDR), NK02 (gentamicin resistant), NK07 (5MDR), UDRT09 (4MDR), and CNRT12 (sensitive to all tested drugs except beta-lactam antibiotics), were subjected to genome sequencing. Genomic DNA of these isolates was extracted by the Wizard Genomic DNA purification kit prior to RNaseA treatment to minimize RNA contamination (Promega Corporation, Madison, WI, United States). The integrity of the genomic DNA was determined by 1% agarose gel electrophoresis, whereas DNA purity and concentration were evaluated by the OD_260__/__280_ spectrophotometer and Qubit dsDNA BR Assay Kit Fluorometric Quantitation (Invitrogen, Carlsbad, CA, United States), respectively. Paired-end libraries were prepared using the NEBNext^®^ Ultra^TM^ DNA Library Prep Kit for Illumina^®^ and genome sequencing was performed using an Illumina HiSeq instrument with the read length of 150 bp.

### Genome Assembly and Annotation

Adaptor sequences and low-quality bases (Q score < 25) were trimmed from raw reads using the Trimmomatic ver. 0.32 (Bolger et al., 2014). The improvement of the read quality was determined using the FastQC ver. 0.11.8.^1^ Trimmed reads were assembled into contigs by the SPAdes ver. 3.13.0 software and assembly quality was verified by the QUAST web service^2^ (Bankevich et al., 2012). To construct the scaffold, contigs were aligned to the reference genome (*A. veronii* B565, GCA_000204115.1) using the Medusa server.^3^ To improve the genome assembly quality, trimmed reads were mapped to the obtained scaffolds by the BWA software^4^ and gaps were filled using Pilon and GMcloser, sequentially (Li and Durbin, 2010; Walker et al., 2014; Kosugi et al., 2015). The assembled genomes were annotated automatically by the NCBI Prokaryotic Genome Annotation Pipeline (PGAP) when the assembled genomes were submitted to the NCBI whole genome shotgun (WGS) web portal (Tatusova et al., 2016). The genomes were assigned the accession numbers GCA_012029595.1 (UDRT09), GCA_012029535.1 (CNRT12), GCA_012029545.1 (NK01), GCA_012029575.1 (NK02), and GCA_012029585.1 (NK07).

### Genome-Based Phylogenetic Analysis

The genome-level taxonomic identification was conducted by submitting the assembled genomes to the Type Strain Genome Server^5^ in which the closest species was determined via the MASH algorithm (Meier-Kolthoff and Goker, 2019). Additionally, the average nucleotide identity (ANI) of the newly assembled genomes in comparison with *A. veronii* biovar sobria [strain 312M (GCA_003859745.1) and LMG13067 (GCA_000820385.1)] and biovar veronii [strain CECT4257 (GCA_000820225.1), CCM4359 (GCA_001908535.1), and C198 (GCA_013697145.1)] was calculated using the ANI calculator^6^ with the ANI cut-off value ≥ 95% (Jain et al., 2018).

Phylogenetic analysis of the newly assembled genomes and other reference strains (*n* = 15, Table 2) was carried out. The sequence type (ST) of the *A. veronii* genomes was classified in comparison with the PubMLST database using the Sequence Query tool provided in the PubMLST website (Jolley et al., 2018). The MLST scheme for *Aeromonas* spp. included the *gltA*, *groL*, *gyrB*, *metG*, *ppsA*, and *redA* genes. The DNA sequence of each locus was exported from the PubMLST: Sequence Query output page. Multilocus sequence analysis (MLSA) was conducted in the PhyloSuite v1.2.1 program based on the DNA sequence variation among these loci (Zhang et al., 2020). Briefly, a multiple sequence alignment of each locus was performed using MAFFT (Katoh and Standley, 2013). All aligned sequences were then concatenated into a contig, and the maximum likelihood tree was constructed using IQ-tree (Nguyen et al., 2015) under the TIM2 + R3 + F model for 5,000 ultrafast bootstraps (Minh et al., 2013). Herein, *Aeromonas schubertii* WL1483 (accession no. NZ_CP013067) was used as the outgroup for MLSA. The consensus tree was visualized using the MEGA X software (Kumar et al., 2018). A total of 14 *gyrB* sequences were retrieved from the *A. veronii* genomes and were used to generate the phylogenetic tree separately from the MLST tree. *Aeromonas schubertii* was used as an outgroup. Phylogenetic analysis was performed using the Molecular Evolutionary Genetic Analysis X (MEGA X) software (Kumar et al., 2018). The Maximum likelihood tree was constructed under the Tamura 3-parameter + GI model with 1,000 replicates. Moreover, a genome-level phylogenetic analysis was also performed based on the sequence variation in the core genome, also called genome-wide SNPs. Core genome among the distinct *A. veronii* genomes was detected and SNPs were called/concatenated/aligned using the automated web-tool CSI Phylogeny v1.4 using the default setting (Kaas et al., 2014). Aligned SNP sequence was used for the maximum likelihood tree reconstruction via the PhyloSuite program as described previously.

**TABLE 2:** List of *Aeromonas veronii* genome included in the resistome analysis.

**Genome completeness**	**Accession no.**	**Strain**	**Host**	**Country**	**Genome size (Mb)**	**Year**
**Complete**	GCA_001634345.1	CB51	Grass carp, *Ctenopharyngodon idella*	China	4.58	2016
	GCA_001593245.1	TH0426	Yellowhead catfish, *Tachysurus fulvidraco*	China	4.92	2016
	GCA_002803925.1	X11	Wuchang bream, *Megalobrama amblycephala*	China	4.28	2017
	GCA_002803945.1	X12		China	4.77	2017
	GCA_003722175.1	MS1837	Catfish, *Siluriformes sp.*	United States	4.68	2018
	GCA_003491365.1	17ISAe	Discus, *Symphysodon discus*	Korea	4.66	2018
**Scaffold**	GCA_002339005.1	UBA1835	European eel, *Anguilla Anguilla*	Spain	4.11	2017
	GCA_003345755.1	XHVA2	Channel catfish, *Ictalurus punctatus*	China	4.91	2018
**Contig**	GCA_000409545.1	PhIn2	Unknown	India	4.30	2013
	GCA_001748325.1	Ae52	Goldfish, *Carassius auratus*	Sri Lanka	4.56	2016
	GCA_002906945.1	ML09123	Catfish, *Siluriformes sp.*	United States	4.75	2018
	GCA_003611985.1	MS1788	Catfish, *Siluriformes sp.*	United States	5.18	2018
	GCA_003367145.1	NS	European bass, *Dicentrarchus labrax*	Greece	4.71	2018
	GCA_003367095.1	VCK	Unpublished	Greece	4.63	2018
	GCA_003036425.1	XHVA1	Channel catfish, *Ictalurus punctatus*	China	5.36	2018
	**GCA_012029595.1**	**UDRT09**	**Hybrid red tilapia, *Oreochromis mossambicus* × *Oreochromis niloticus***	**Thailand**	**4.61**	**2018**
	**GCA_012029535.1**	**CNRT12**		**Thailand**	**4.90**	**2018**
	**GCA_012029545.1**	**NK01**	**Nile tilapia, *Oreochromis niloticus***	**Thailand**	**4.56**	**2015**
	**GCA_012029575.1**	**NK02**	**Nile tilapia, *Oreochromis niloticus***	**Thailand**	**4.80**	**2015**
	**GCA_012029585.1**	**NK07**	**Nile tilapia, *Oreochromis niloticus*)**	**Thailand**	**4.78**	**2015**

*Bold represents the newly sequenced genomes used in this study, while the other genomes were obtained from the GenBank database.*

### Resistome Analysis

The *A. veronii* genomes listed in Table 2 were also used for the resistome analysis. The genomes were queried against the Comprehensive Antibiotic Resistance Database (CARD^7^) (Alcock et al., 2019) for the *in silico* prediction of the possible antimicrobial resistance gene (ARGs) contained in the genome. Resistance Gene Identifier (RGI) automate tool was used for the gene analysis in CARD. The search criteria were set as “perfect and strict hits only” with a “high quality/coverage” sequence quality. Nucleotide identity to query sequences lower than 96% were excluded (Lomonaco et al., 2018). In addition, the presence of acquired antimicrobial resistance genes in the *A. veronii* genomes were also analyzed through ResFinder V3.1^8^ (Bortolaia et al., 2020). Genome (fasta file) was uploaded to the web resource with a 95% identity of sequence threshold and 80% minimum length of alignment setting (Lomonaco et al., 2018). The amino acid sequences of the predicted ARGs were downloaded from CARD and ResFinder and were used to construct the local blast database on the Blast2GO software in order to perform a reciprocal BLAST. The deduced amino acid sequences (proteome) from each *A. veronii* isolate of this study were queried against the local blast database using blastp implemented in the Blast2GO program (Conesa et al., 2005).

### Genomic Islands Analysis

The presence of genomic island (GI) and resistance island (RI) within the newly sequenced *A. veronii* genomes (NK01, NK02, NK07, CNRT12, and UDRT09) were predicted using IslandViewer4^9^ (Bertelli et al., 2017). The GenBank file was uploaded to the IslandViewer4 web interface in which *A. veronii* 17ISAe was selected as a reference genome for the alignment. GI was predicted using the IslandPick, SIGI-HMM, and IslandPath-DIMOB methods (Bertelli et al., 2017) implemented in IslandViewer. The output was manually compared to the complete *A. veronii* genomes (strain CB51, TH0426, X11, X12, MS1837, and 17ISAe).

## Results

### Taxonomic Identification

According to the partial *gyrB* sequence (1.4-kb-long), all bacterial isolates were mostly similar to *A. veronii* with a 98%–99% sequence identity (100% query coverage). Likewise, the TYGS web tool also indicated that the representative genomes of this study (NK01, NK02, NK07, CNRT12, and UDRT09) are genotypically closest to *A. veronii* CECT 4257. The digital DNA-DNA hybridization (dDDH) value of the current genomes in comparison with *A. veronii* CECT 4257 is 69.18% (d_4_ formula) and the GC% difference is 0.28. The dDDH cut-off for the bacterial species demarcation is ≥70%. Thus, the Aeromonad isolates used in this study should be classified as *A. veronii*. Herein, the type strain *A. veronii* CECT 4257 belongs to biovar veronii. This study also attempted to identify the biovar of the current isolates based on a pairwise genome comparison against both biovars of *A. veronii*, namely biovar sobria and biovar veronii. The ANI calculator revealed that the genomes of this study were similar to both biovar sobria (ANI = 96.05%–96.45%) and biovar veronii (ANI = 96.16%–96.48%). The ANI values in comparison with both biovars are demonstrated in the supplementary information (Supplementary Table 1). Since the ANI cut-off for species demarcation is ≥95%, we cannot differentiate the biovar of the current *A. veronii* isolates based on their genomes alone. However, there was evidence that biovar veronii could be differentiated from biovar sobria by the exhibition of ornithine decarboxylase negative and arginine dihydrolase positive activities (Abbott et al., 2003). According to the biochemical profiles shown in Supplementary Table 2, the recent isolates shared a similar characteristic with the isolates determined in our previous study (Dong et al., 2015). Thus, with reference to the biochemical profile, *A. veronii* in the present study can be distinguished as biovar veronii.

### Determination of the Minimum Inhibitory Concentration

The broth microdilution method was used to evaluate the MIC and the results are demonstrated in Table 3. The results suggested that the isolates of this study were completely resistant to beta-lactams (ampicillin and amoxicillin; MIC > 256 mg/L). On contrary, all isolates were susceptible to florfenicol with the MIC lower than 1 mg/L. The percentage of resistance to other antimicrobials ranged from oxytetracycline (67%) to oxolinic acid (33%) and enrofloxacin (17%). More importantly, this research found the first resistance of *A. veronii* isolated from freshwater fish to gentamicin and Trimethoprim/sulfamethoxazole (MIC > 256 mg/L) in the NK02 and NK07 isolates, respectively. Furthermore, the isolates with a diverse degree of multidrug resistance (MDR), namely NK07 (six drugs from five antimicrobial classes, 5MDR), UDRT09 (four antimicrobial classes, 4MDR), NK01 (three antimicrobial classes, 3MDR), NK02 (gentamicin resistant), and CNRT12 (resistant to only beta-lactam, Sense), were included in the subsequent genome investigation.

**TABLE 3:** MIC values *Aeromonas veronii* of this study.

**Antimicrobial**		Cut-off value*	**MIC (mg/L)**											
**Class**	**Drug**		**NK01**	**NK02**	**NK03**	**NK04**	**NK05**	**NK06**	**NK07**	**CNRT07**	**CNRT11**	**CNRT12**	**CNRT13**	**UDRT09**
Beta-lactam	Amoxicillin	NA	> 256^*R*^	>256^*R*^	> 256^*R*^	>256^*R*^	> 256^*R*^	>256^*R*^	> 256^*R*^	>256^*R*^	> 256^*R*^	>256^*R*^	> 256^*R*^	>256^*R*^
	Ampicillin	NA	> 256^*R*^	>256^*R*^	> 256^*R*^	>256^*R*^	> 256^*R*^	>256^*R*^	> 256^*R*^	>256^*R*^	> 256^*R*^	>256^*R*^	> 256^*R*^	>256^*R*^
Aminoglycoside	Gentamicin	4	2	> 256^*R*^	2	8	4	2	4	2	4	2	2	4
Fluoroquinolone	Enrofloxacin	0.125	2	0.5	2	1	< 0.03	1	4 ^*R*^	0.5	0.5	< 0.03	0.5	> 16 ^*R*^
Quinolone	Oxolinic acid	0.03	64 ^*R*^	1	32 ^*R*^	1	< 0.25	8	32 ^*R*^	2	8	< 0.25	4	64 ^*R*^
Tetracycline	Oxytetracycline	0.25	> 256^*R*^	1	>256^*R*^	> 256^*R*^	1	128 ^*R*^	> 256^*R*^	1	128 ^*R*^	1	128 ^*R*^	> 256^*R*^
Sulfonamide	Sulfamethoxazole/Trimethoprim	0.25	4	2	2	2	2	2	> 256^*R*^	2	2	2	2	2
Phenicol	Florfenicol	2	1	1	1	1	1	1	1	1	1	1	1	1
Interpretation			3MDR		3MDR		Sense		5MDR			Sense		4MDR

*^∗^The cut-off referred to the generic epidemiological cut-off values of *Aeromonas* sp. in freshwater Baron et al., 2017.*

*Bold represents the isolates used for whole-genome sequencing. Superscripts R indicated a resistance. NA, not applicable.*

### Phylogenetic Analysis

**FIGURE 1:**
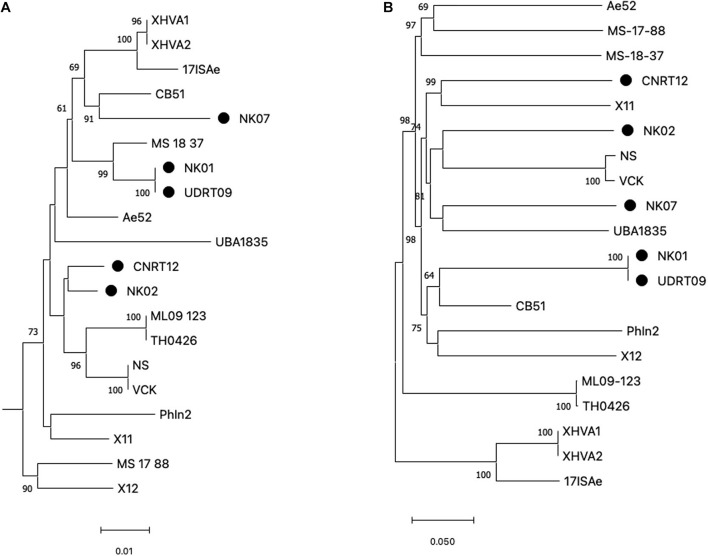
Maximum likelihood tree generated from concatenated MLST loci **(A)** and genome-wide SNPs **(B)**. MLST loci included *gltA*, *groA*, *metG*, and *recA* with the total length of 2,070 bp and *Aeromonas schubertii* WL1483 as an outgroup (omitted from the tree). The genome-wide SNPs was concatenated into contig with a total length of 175,597 bp. Trees were constructed using IQ-tree with 5,000 ultrabootstrap. Numbers at the tree node represent the bootstrap value in percentage (only ≥ 60 value is shown). The newly assembled genomes (Thai isolates) are indicated by the filled circle adjacent to the taxa. Scale bar represents the nucleotide substitution per site.

Among the 20 *A. veronii* genomes used for the genome analysis, ST could be assigned to only 4 genomes, i.e., MS1837 (ST 254), 17ISAe (ST485), NS, and VCK (ST 23). On the other hand, the other isolates were defined as an unknown ST due to the presence of new allele variants (Supplementary Table 2). In this study, only four MLST loci (*gltA*, *groA*, *metG*, and *recA*), with a total length of 2,070 bp, were included for the phylogenetic tree reconstruction since *gyrB* and *ppsA* were absent in some isolates due to the incompleteness of the genomes. However, since *gyrB* is a key genetic marker for *Aeromonas* species identification, a phylogenetic analysis of *gyrB* was separately performed to compare with the MLST loci. The results from both the *gyrB*-based tree and MLSA-based tree suggested a high genetic diversity among the *A. veronii* isolates (Figure 1A and Supplementary Figure 2). The observed phylogeny was likely unrelated to the geographical origin, host species, and resistance phenotype of the analyzed taxa. For instance, the isolates from Thailand are clustered in distinct clade disperse, whereas the isolates NK02 and CNRT12 were grouped into the same subclade regardless of the difference in the resistance phenotype (aminoglycoside resistant vs. sensitive). Similarly, the phylogenetic tree based on the genome-wide SNPs also suggested a high genetic variability (Figure 1B). The numbers of core genome SNPs (shown in the Supplementary Figure 1) of the pairwise comparison across the *A. veronii* genomes were diverse with between 11 and 45,536 SNPs. It is worth mentioning that two out of five newly assembled genomes (NK01 and UDRT09) were almost genetically identical (only 27 SNPs) and have similar resistance phenotypes (resistant to quinolone, tetracycline, and beta-lactam). This indicated that NK01 and UDRT09 were derived from the same clone.

### Resistome Analysis

*Aeromonas veronii* genomes were queried against two AMR databases, namely CARD and ResFinder, to identify the ARG repertoire, also known as resistome. The bacteria used in this analysis comprised of (i) the strains isolated from tilapia and (ii) other freshwater fishes whose genomes were retrieved from the NCBI (Table 2). The potential ARGs observed in each group of *A. veronii* strains were described as follows:

#### Resistome of *Aeromonas veronii* Isolated From Tilapia

**FIGURE 2:**
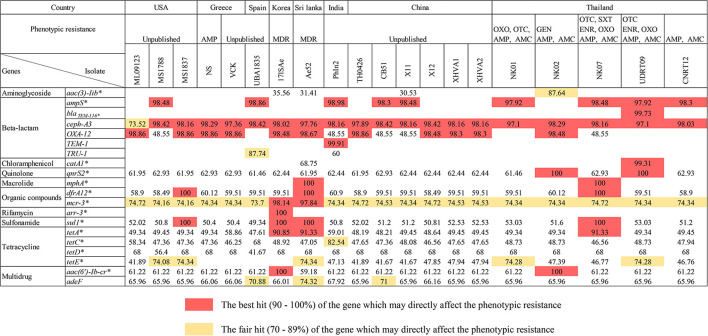
The presence of ARGs within the genomes of *A. veronii* isolated from freshwater fishes (*n* = 20). The numbers represent the pairwise percent identity (obtained from protein BLAST) using the deduced amino acid sequence and its homolog as query and subject sequence, respectively. High percent identity is suggested by a darker color, whereas an empty box indicated that no homologous gene is observed in either the CARD or ResFinder database. Strains isolated from tilapia (newly sequenced genomes) are defined by an underlined name. Acquired antimicrobial resistant genes are labeled with an asterisk.

There were 17 ARGs identified from the newly assembled genomes derived from tilapia (*n* = 5; NK01, NK02, NK07, UDRT09, and CNRT12). These ARGs can be categorized into nine groups according to the antimicrobial classes, i.e., aminoglycoside, beta-lactam, chloramphenicol, macrolide, organic compound, quinolone, sulfonamide, tetracycline, and multidrug resistance-associated (Figure 2). Among the five isolates, UDRT09 and NK07 carried the greatest number of ARGs (14 genes), while NK02, NK01, and CNRT12 carried 13, 12, and 12 genes, respectively. Of these 17 ARGs, 11 genes [*ceph-A3*, *qnr32*, *dfrA12*, *mcr-3*, *sul1*, *tetA*, *tetC*, *tetD*, *tetE*, *aac*(*6’*)*-Ib-cr*, and *ade-F*] were shared by all *A. veronii* tilapia isolates. Herein, two ARGs associated with macrolide resistant (*mphA*), aminoglycoside resistant (*aac*(*3)-IIb*), and chloramphenicol (*catA1*) were specifically found in NK07, NK02, and UDRT09, respectively.

#### Resistome of *Aeromonas veronii* Isolated From Other Freshwater Fishes

A total of 19 ARGs (ten drug classes) were identified from 15 genomes of *A. veronii* associated with an invasive disease in various freshwater fish species (Figure 2). The genomes from PhIn2 and Ae52 (isolated from India and Sri Lanka) harbored the highest number of ARGs (15 genes), followed by X11 (China), CB51 (China), 17ISAe (Korea), and MS1788 (United States) which carried 14, 13, 13, and 13 ARGs, respectively. The strains UBA1835 (Spain); ML09123 and MS1837 from United States; and TH0426, X12, XHVA1, and XHVA2 from China each carried 12 ARGs. Lastly, 11 ARGs were detected in VCK and NS from Greece. There was one strain, namely 17ISAe, that carried the rifamycin resistance gene (*arr*-3) which was absent in other *A. veronii* isolates, including the tilapia isolates.

Concerning the sharing of ARGs among the Aeromonad population, there were 16 ARGs detected in both tilapia and freshwater fish isolates (*n* = 20). The following genes: *aac* (6′)-*lb-cr*, *adeF*, *cphA3*, *qnrS2*, *drf A12*, *mcr-*3, *sul-1*, *tetA*, *tetC*, and *tetD* were detected in all isolates. The *OXA*-12 gene was observed in only two out of five tilapia isolates (NK02 and NK09), but abundant in the strains isolated from other fish species (14 out of 15). This was similar to the absence of *tetE* in only four isolates from freshwater fish. In addition, *catA1* and *mphA* were identified in one strain, i.e., Ae52, similar to the gene observed in UDRT09 and NK07 from the tilapia group.

### Genomic Islands Analysis

**FIGURE 3:**
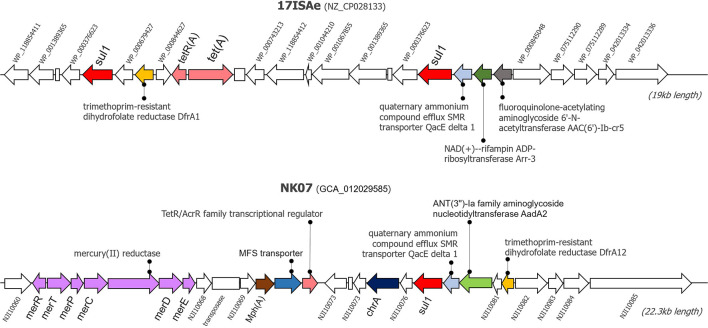
Graphical representation of resistance islands presented in the genome of *A. veronii* 17ISAe and NK07. These resistance islands were predicted using the IslandPath-DEMOB and SIGI-HMM methods on the IslandViewer 4 web server. Arrows represents the coding sequences, whereas an empty box indicates a pseudogene. CDS accession numbers or gene name are described adjacent to the gene. Antimicrobial and heavy metal resistance genes are labeled by color.

Since the newly sequenced genomes used for GI detection are incomplete genomes, any predicted GI located near the boundary of contigs (< 1,000 bp from 5′ and 3′ termini) were omitted from this analysis to avoid a false positive detection. Herein, IslandViewer 4 predicted the presence of potential GIs in every tilapia *A. veronii* isolate genome. A manual investigation of the gene contents resided within these potential GIs showed that the NK07 isolate harbored a large resistance island (RI) with a size of 22,323 bp (Figure 3). This RI contained several AMRs and heavy metal resistance genes (MRGs) [mercury resistance (*mer*) operon and chromate (*chrA*) efflux pump]. There were at least six AMRs potentially associated with the resistant to diverse drug classes including sulfonamide (*sul1*), trimethoprim (*drfA12*), tetracycline (*tetR/acrR*), aminoglycoside (*aadA2*), macrolide (*mphA*), and multidrug efflux pump (MFS transporter). Additionally, this NK07-derived RI contained one antiseptic resistance gene (*qacE*). Interestingly, some resistance elements, i.e., *sul1*, *qacE*, and trimethropim-resistant dihydrofolate reductase (*dfrA*), were also observed in an important 19-kb-long RI presented in the reference strain 17ISAe (Figure 3), although other genetic contents were dissimilar to the NK07-derived RI. Regarding the other *A. veronii* tilapia isolates, the 12-kb-long RI containing tetracycline resistance genes (*tetE* and *tetR*) was identified in the genome of the NK01 and UDRT09 isolates (Supplementary Figure 3), while no GI carrying AMRs was observed in the NK02 and CNRT12 isolates.

## Discussion

Aeromonad infection is one of the global concerns to the loss of aquaculture production. Due to the rise of antimicrobial resistance (AMR), the solution and choice of antimicrobial use is a challenge to the farmer and researcher. In this study, *Aeromonas* spp. were isolated from tilapia and according to our findings, these were classified as *A. veronii* biovar veronii based on their biochemical characterization (Abbott et al., 2003).

Resistome analysis is capable of investigating the comprehensive data of AMR to improve the efficacy of antimicrobial use (Zankari et al., 2012). As a result, in the MIC assay, florfenicol showed the best efficacy against *A. veronii.* However, the isolates in this study were altogether resistant to beta-lactams (amoxicillin and ampicillin) with a high MIC value, similar to that reported in the previous publication (Janda and Abbott, 2010; Yang et al., 2017). Referring to the epidemiological cut-off values from the study of *Aeromonas* diversity in France (Baron et al., 2017), the MIC values of *A. veronii* in this study were seriously higher than the previous publication. Likewise, this is the first discovery of gentamicin and sulfamethoxazole/trimethoprim resistant *A. veronii* isolated from tilapia. These rare resistances were reported previously in the clinical and environmental samples from Australia where *Aeromonas* spp. was susceptible to gentamicin and sulfamethoxazole/trimethoprim, with more than 98% and 99%, respectively (Aravena-Roman et al., 2012).

According to the licensed antimicrobials allowed for use in Thailand (FCSTD, 2012), oxytetracyclines are commonly used in tilapia farming which promotes a high resistance as evidenced by the MIC values that were higher than those in the previous report (Skwor et al., 2014). In addition, oxolinic acid and enrofloxacin were both less effective against *A. veronii*, in contrast to the study from China whereby *A. veronii* isolated from Chinese long-snout catfish was susceptible to these antimicrobials (Cai et al., 2012). Besides, *A. veronii* isolated from tilapia revealed a resistance to multiple antimicrobials similar to the study in channel catfish (Yang et al., 2017). MDR was observed mainly in NK07 (5MDR) followed by UDRT09 and NK01, which are 4MDR and 3MDR, respectively.

Regarding the ARGs, their presence and transmission were implicated in the efficacy of human and animal diseases treatment caused by the resistant *A. veronii* (Yang et al., 2017). The resistome analysis of the recent *A. veronii* isolates from tilapia and other freshwater fish isolates was performed by the consideration of the ARGs associated with a phenotypic resistance expression. It revealed 20 ARGs, which belong to nine groups of antimicrobial classes as shown in Figure 2. Overall, the ARGs were shared among the isolates from both tilapia and other fish species. The reciprocal protein BLAST against CARD-derived ARGs sequences indicated a relatively high percent identity (over 80%) suggesting a potentially complete or functional genes in this study.

### Aminoglycoside Resistome

The resistant phenotype observed in NK02 was also in accordance with the presence of the potentially completed aminoglycoside resistance genes [*aac* (3)-*IIb* and *aac* (6′)-*Ib*-*cr*] exclusively found in its genome. Notably, there were few reports of aminoglycoside resistance in *Aeromonas* sp. from channel catfish and discus (Yang et al., 2017; Roh et al., 2019). In any case without evidence of drug use before, ARGs may then be acquired from the other pathogens or surrounding the environment (Heuer et al., 2002).

### Beta-Lactam Resistome

Beta-lactams are broadly used worldwide; however, the recent *A. veronii* isolates were resistant to both amoxicillin and ampicillin categorized as broad-spectrum beta-lactams. The set of beta-lactam resistance genes were detected in all isolates which are typically found in the *Aeromonas* species (Chen et al., 2019). Previously, *A. hydrophila* and other species of *Aeromonas* have been reported as intrinsically ampicillin-resistant (Joseph et al., 1979; Yucel and Aslim, 2005). However, there is no official report of intrinsic resistance in *A. veronii*. Referring to the MIC values that were supported by the presence of beta-lactams resistance-associated genes (*ampS, ceph*-*A3* and *OXA*-*12*) with over a 97% sequence identity, *A. veronii* should be noted as an intrinsically broad-spectrum beta-lactam resistant as mentioned in the previous publications (Baron et al., 2017). Extended spectrum beta-lactamases (ESBLs) are enzymes which enable bacteria to hydrolyze an extended spectrum cephalosporin (Ghafourian et al., 2015). So far, there are no reports about ESBL drug group use in Thai aquaculture. However, the ESBL-related gene detected in this study was probably acquired from agricultural, human medication, or other sources as reported from a previous publication (Piotrowska et al., 2017). Further investigation of the phenotypic susceptibility to ESBL is needed.

### Quinolone and Fluoroquinolone Resistome

The presence of the *qnrS2* gene refers to the plasmid-mediated quinolone resistance protein which was originally found in *Salmonella enterica* and plays a role on horizontal gene transfer (Jia et al., 2017). This gene was detected in all isolates but only showed a perfect identity in NK02 and UDRT09. However, there were few publications which reported that *A. veronii* encoded the *qnrS2* gene on a plasmid since the first report in 2008 (Sanchez-Cespedes et al., 2008). Generally, missense mutations in DNA gyrase (*gyrA* or *gyrB*) and topisomerase IV (*parC* or *parE*) encoded genes are common mechanisms that confer a resistance to fluoroquinolones while *qnrS2* plays a role as a supportive resistance gene (Sanchez-Cespedes et al., 2008; Redgrave et al., 2014).

### Tetracycline Resistome

Five ARGs were blasted against the *A. veronii* isolates, and these included the *tetA*, *tetC*, *tetD*, *tetE*, and *adeF* genes. The *adeF* works as a secondary resistance gene and enhances tetracycline and fluoroquinolone resistance (Mobasseri et al., 2018). In addition, a set of *tet* genes are located on a plasmid and functionally work for tetracycline resistance (Jia et al., 2017). Similar to the previous study, *A. veronii* resistance to tetracycline and their associated genes have been reported worldwide (Skwor et al., 2014; Baron et al., 2017; Yang et al., 2017). The set of *tetA*, *tetC*, *tetD*, *tetE*, and *adeF* genes were found in most of the isolates. As seen in the NK01, NK07, and UDRT09 isolates, the involvement of *tet* genes (high percent identity) coincide with higher MIC values as mentioned in the previous publication (Balassiano et al., 2007).

### Sulfonamide Resistome

A sulfonamide resistance gene (*sul1*) is a gene encoding dihydropteroate synthase and it was reported as multidrug resistance mediated by class 1 integrons in *Aeromonas* (Deng et al., 2016). The gene was presented in all strains used in this analysis, although only four strains (MS1788, 17ISAe, Ae52, and NK07) presented a 100% sequence identity. Interestingly, the presence of a complete *sul1* in NK07 consistent to its Sulfamethoxazole/Trimethoprim resistance phenotype was indicated by the MIC assay.

### Other Resistance Genes

The group of class 1 integron resistance association consists of genes *catA1* and *dfrA12*. These genes are acquired differently from other pathogens. Generally, *catA1* is a gene encoding chloramphenicol acetyltransferase from *Shigella flexneri* 2a and *dfrA12* is a gene encoding dihydrofolate reductase from *Vibrio cholera* (Jia et al., 2017). Similar to this study, *catA1* was detected in *A. salmonicida* and recently in *A. veronii* (Tanaka et al., 2016; Syrova et al., 2018). In addition, *dfrA12* was reported as MDR mediated by class 1 integrons in *Aeromonas* isolates (Deng et al., 2016). However, the effect of the high identity of *catA1* and *dfrA12* gene to the phenotypic resistance was not evaluated in UDRT09 and NK07. Lastly, *mcr-3*, a transferable colistin resistance gene which was firstly isolated from the pWJ1 plasmid of *E. coli*, showed a high amino acid identity to the *Aeromonas* species in this study (Yin et al., 2017). However, an evaluation of the colistin MIC value to confirm the potential contribution of the gene to phenotypic resistance was not performed in the current study.

### Genomic Islands Analysis

Genomic Islands analysis enables the prediction of the potential resistance elements known as the Horizontal Gene Transfer (HGT) foundation (Soares et al., 2016). This study analyzed the potential of resistance and GIs association as clearly seen on the NK07 resistance island (NK07-RI). First of all, NK07-GIs are larger than 17ISAe (MDR isolate from discus) and much larger when compared to the less resistant isolates (NK01 and UDRT09). ARGs (*sul1*, *drfA12*, *tetR*/*acrR*, *aadA2*, and *mphA*) of NK07-RI had perfect sequence identity hits which are related to high MIC values. These genes have been reported as a mobile genetic element associated, as described before. Multidrug efflux pump (MFS transporter) plays an important role together with the mobile element protein to promote the MDR isolate (Pasqua et al., 2019). Moreover, antiseptic resistance gene (*qacE*) detected in NK07-RI has been studied in many pathogens with the effect of reducing the susceptibility of biocide (antiseptics and disinfectants) and antimicrobials (Vijayakumar and Sandle, 2019). Although, there is no study about this effect in *A. veronii*, the presence of *qacE* and resistance potential should be of concern. As seen in Figure 3, several MRGs were detected in NK07-RI. The chromate efflux pump associated gene (*chrA*) and mercury resistance (*mer*) operon are both generally encoded on a plasmid which mainly works on cell detoxify mechanism (Boyd and Barkay, 2012; Baaziz et al., 2017). Previously, *Mer* operon was detected in MDR *A. veronii* strain MS-18-37 isolated from United States catfish but no study about its function was demonstrated (Abdelhamed et al., 2019). Due to the ability of heavy metal uptake mechanism, *Aeromonas* spp. was tested as a bioremediation property for wastewater treatment (Ogugbue and Sawidis, 2011). This finding represents an adaptation ability to selective pressure in a microorganism induced by heavy metal residues contaminated in a water source. Although, there are several studies of MRGs with ARGs, their functions remain unclear (Chen et al., 2019).

## Conclusion

Resistome analysis of *A. veronii* isolated from tilapia in Thailand provided evidence that conventional antimicrobials used in aquaculture are going to lose their effectiveness. According to the licensed antimicrobials allowed for use in Thailand, amoxicillin, oxytetracycline, and oxolinic acid may not be recommended for a longer use, likewise, enrofloxacin requires a high dosage for its usage (more than 16 mg/L) but there should be concern about the effect of resistant *A. veronii* in human medication. The last choice of the recommended antimicrobial use is sulfamethoxazole/trimethoprim and florfenicol (after license announcement by the FDA). In this study, *A. veronii* isolates were adapted into a multidrug-resistance related to the presence of multiple ARGs, and several genes were shared in the aquatic system among the *A. veronii* population worldwide. The prevalence of resistance against ESBL, beta-lactam, and colistin in *A. veronii* is highlighted and requires more insight study. Moreover, the possibility of plasmid-mediated resistance gene acquisition especially in gentamicin and sulfamethoxacin should be of concern as these can affect human and other animal health care. It should be noted that *A. veronii* has a broad-spectrum beta-lactam intrinsic resistance as revealed by the current and previous studies. At last, the outcomes of this study can be applied for AMR prediction and further treatment plans for effective antimicrobial use.

## Data Availability Statement

The datasets presented in this study can be found in online repositories. The names of the repository/repositories and accession number(s) can be found below: https://www.ncbi.nlm.nih.gov/bioproject/PRJNA612772.

## Author Contributions

RS, PC, ND-H, and PK: data curation. RS and PK: investigation and writing of the original draft. IH and CR: resources. PK and CR: supervision and validation. RS, PC, ND-H, ES, PK, RC, and CR: writing—review and editing. CR: funding acquisition. All authors contributed to the article and approved the submitted version.

## Conflict of Interest

The authors declare that the research was conducted in the absence of any commercial or financial relationships that could be construed as a potential conflict of interest.

## Publisher’s Note

All claims expressed in this article are solely those of the authors and do not necessarily represent those of their affiliated organizations, or those of the publisher, the editors and the reviewers. Any product that may be evaluated in this article, or claim that may be made by its manufacturer, is not guaranteed or endorsed by the publisher.

## Footnotes

1
http://www.bioinformatics.babraham.ac.uk/projects/fastqc/

2
http://quast.bioinf.spbau.ru/

3
http://combo.dbe.unifi.it/medusa

4
http://bio-bwa.sourceforge.net/

5
https://tygs.dsmz.de/

6
https://www.ezbiocloud.net/tools/ani

7
http://arpcard.mcmaster.ca/

8
http://cge.cbs.dtu.dk/services/ResFinder

9
https://www.pathogenomics.sfu.ca/islandviewer
