# Supplementary material for: Insight Into Whole Genome of Aeromonas veronii Isolated From Freshwater Fish by Resistome Analysis Reveal Extensively Antibiotic Resistant Traits
Source: Front Microbiol. 2021 Sep 17;12:733668. doi: 10.3389/fmicb.2021.733668 (PMC8484913; doi:10.3389/fmicb.2021.733668)
Supplement: Supplementary file 1 [file Data_Sheet_1.PDF]

Supplementary Material

1 Supplementary Tables

Table S1 The pairwise average nucleotide identity (ANI) values between the newly sequenced genomes (*n* = 5) in comparison to *Aeromonas veronii* biovar sobria and biovar veronii. Genbank accession numbers are provided in parenthesis.

Genome of this study	Reference *Aeromonas veronii* genomes				
	bv.sobria		bv.veronii		
	312M (GCA_003859745)	LMG13067 (GCA_000820385)	C198 (GCA_013697145)	CCM4359 (GCA_001908535)	CECT4257 (GCA_000820225)
UDRT09	96.14	96.19	96.18	96.33	96.24
CNRT12	96.2	96.45	96.26	96.43	96.46
NK01	96.07	96.21	96.14	96.35	96.32
NK02	96.09	96.39	96.31	96.31	96.25
NK07	96.05	96.37	96.16	96.48	96.5

Table S2 Biochemical profile of five *A. veronii* recent isolates with three isolates from previous study and two reference isolate in difference biovar.

Biochemical test	Recent isolates					Previous study (Dong et al., 2015)		Reference isolate (Abbott et al., 2003)	
								A. ***veronii***	
	CNRT07	CNRT11	CNRT12	CNRT13	UDRT09	NK01	NK02, NK07	bv.veronii	bv.sobria
Morphology	Gram negative short rod-shape								
Hemolysis	A	B	B	B	B	B	A	+	+
Oxidase	+	+	+	+	+	+	+	+	+
Catalase	+	+	+	+	+	+	+	+	+
Motility	+	+	+	+	+	+	+	+	+
O/F (Oxidation and Fermentation)	F	F	F	F	F	F	F	ND	ND
Decarboxylase									
Arginine	+	+	+	+	+	+	+	-	+
Lysine	+	+	+	+	+	+	+	+	+
Ornithine	-	-	-	-	-	-	-	+	-
Indole	+	+	+	+	+	+		+	

* The result was interpreted after incubation at 28◦C for 24h, (-); negative, (+); positive,

F; Fermentation, A; alpha-hemolysis, B; beta-hemolysis, ND; non-determine

Table S3 MLST allele and sequence type (ST) identified from the genomes of *Aeromonas veronii* (n = 20) and *A. schubertii* WL1483

	gltA	*groL*	*gyrB*	*metG*	*ppsA*	*recA*	ST
CB51	127					378	ND
TH0426	17	28	29		26	28	ND
X11	358	221	479	227		53	ND
X12	56		466				ND
MS1837	151	216	212	213	84	220	254
17ISAe	354	154	126	16	353	374	485
UBA1835		242					ND
XHVA2	154	126				374	ND
PhIn2							ND
Ae52	229	223	232	46		86	ND
ML09123	17	28	29		26	28	ND
MS1788	110	137	137	131	225	131	ND
NS	25	24	25	24	22	24	23
VCK	25	24	25	24	22	24	23
XHVA1		154	126			374	ND
UDRT09			221				ND
CNRT12	110		221	81		476	ND
NK01			221				ND
NK02		21				11	ND
NK07		319					ND
A.schubertii WL1483	16	251	257	256	274	270	331

Underline represents the newly sequenced genomes of this study. Only exact match allele is shown.

ND, unable to determined due to the unavailability of allele profiles in PubMLST database.

2 Supplementary Figures

	17ISAe	Ae52	CNRT12	ML09-123	MS-17-88	MS-18-37	NK01	NK02	NK07	NS	PhIn2	CB51	TH0426	UBA1835	UDRT09	VCK	X11	X12	XHVA1	XHVA2
17ISAe	0	43554	44020	44062	43623	43912	45451	43896	44057	44469	45020	34886	44140	43606	45450	44231	44002	44393	19748	19745
Ae52	43554	0	43823	43512	42935	43096	45010	43789	44105	44510	44689	34433	43566	43345	45013	44355	43621	44185	43549	43554
CNRT12	44020	43823	0	44121	43954	43486	44776	43594	43937	44123	44635	35157	44202	43025	44779	44029	43025	43729	44033	44026
ML09-123	44062	43512	44121	0	43865	43890	45314	44053	44390	44759	45132	35201	591	43442	45315	44560	44095	44512	42478	42471
MS-17-88	43623	42935	43954	43865	0	43476	45054	43748	44093	44478	44889	34660	43933	43064	45051	44318	43463	44308	43576	43573
MS-18-37	43912	43096	43486	43890	43476	0	44920	43732	43814	44436	44968	35011	43967	43241	44920	44262	43154	43997	43744	43743
NK01	45451	45010	44776	45314	45054	44920	0	44923	45169	45512	45536	35440	45385	44488	27	45434	44774	45284	45355	45346
NK02	43896	43789	43594	44053	43748	43732	44923	0	43696	43769	44795	34841	44110	42976	44924	43550	43485	44025	43769	43772
NK07	44057	44105	43937	44390	44093	43814	45169	43696	0	43956	45229	35068	44476	43041	45166	43790	43634	44292	43974	43967
NS	44469	44510	44123	44759	44478	44436	45512	43769	43956	0	45554	35544	44801	43378	45510	4068	43974	44471	44418	44413
PhIn2	45020	44689	44635	45132	44889	44968	45536	44795	45229	45554	0	35299	45172	44214	45539	45393	44614	45050	44934	44927
CB51	34886	34433	35157	35201	34660	35011	35440	34841	35068	35544	35299	0	35242	34365	35435	35433	34922	35137	34810	34799
TH0426	44140	43566	44202	591	43933	43967	45385	44110	44476	44801	45172	35242	0	43515	45386	44604	44141	44580	42536	42527
UBA1835	43606	43345	43025	43442	43064	43241	44488	42976	43041	43378	44214	34365	43515	0	44487	43249	43086	43526	43543	43536
UDRT09	45450	45013	44779	45315	45051	44920	27	44924	45166	45510	45539	35435	45386	44487	0	45431	44775	45285	45354	45345
VCK	44231	44355	44029	44560	44318	44262	45434	43550	43790	4068	45393	35433	44604	43249	45431	0	43763	44386	44173	44166
X11	44002	43621	43025	44095	43463	43154	44774	43485	43634	43974	44614	34922	44141	43086	44775	43763	0	43759	43941	43938
X12	44393	44185	43729	44512	44308	43997	45284	44025	44292	44471	45050	35137	44580	43526	45285	44386	43759	0	44231	44224
XHVA1	19748	43549	44033	42478	43576	43744	45355	43769	43974	44418	44934	34810	42536	43543	45354	44173	43941	44231	0	11
XHVA2	19745	43554	44026	42471	43573	43743	45346	43772	43967	44413	44927	34799	42527	43536	45345	44166	43938	44224	11	0

Figure S1 Pairwise SNP count matrix. Core genome SNPs were called and counted using the automated CSIPhylogeny v1.4 web server (https://cge.cbs.dtu.dk/services/CSIPhylogeny/)

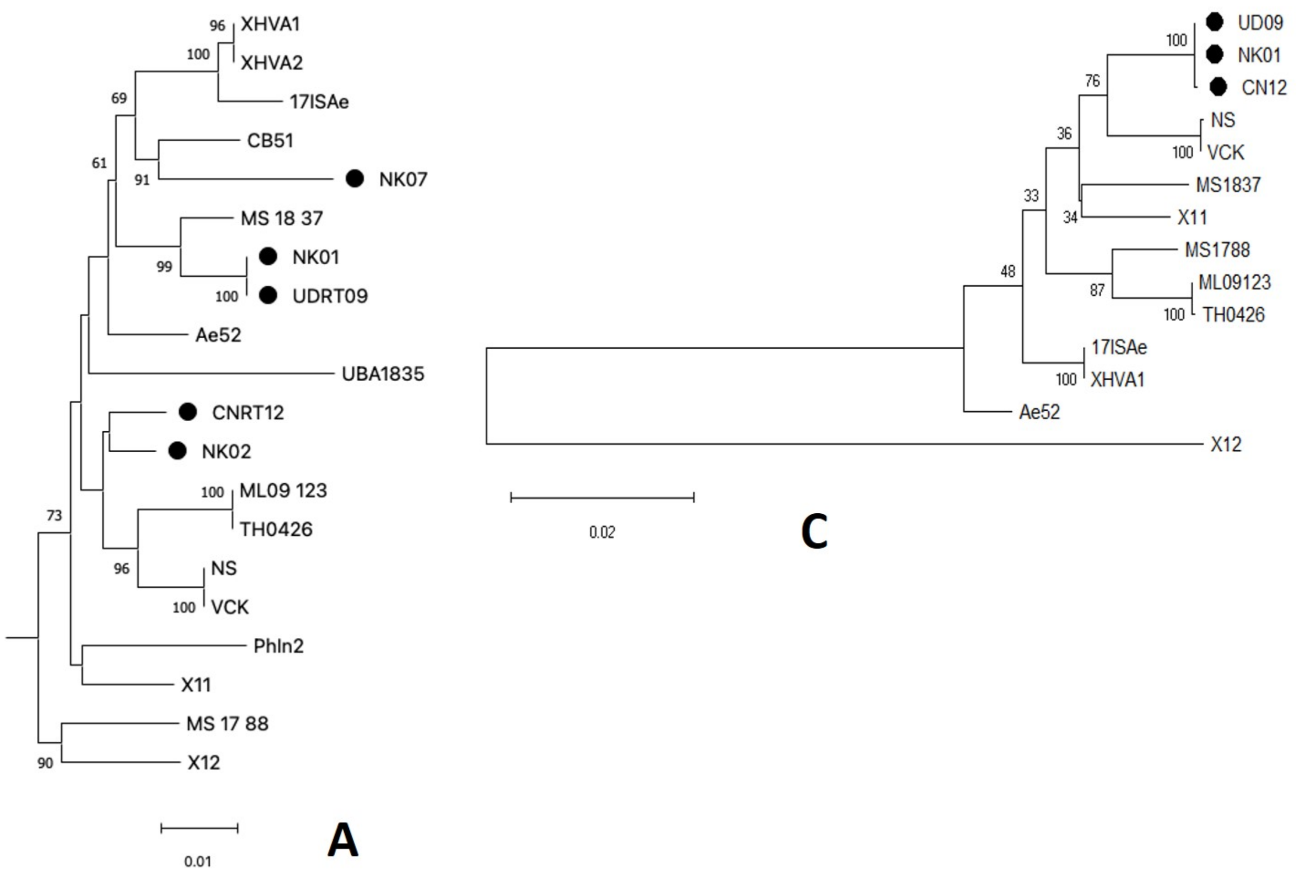

Figure S2 Maximum likelihood tree generated from *gyrB* sequences (C) compare to MLST loci tree (A). The *gyrB* phylogenetic tree was constructed using Tamura 3-parameter +GI model with 1,000 replicates. *Aeromonas schubertii* WL1483 as an outgroup (omitted from the tree). Numbers at tree node represent bootstrap value in percentage (only ≥60 value is shown). The newly assembled genomes (Thai isolates) are indicated by filled circle adjacent to the taxa. Scale bar represents nucleotide substitution per site.

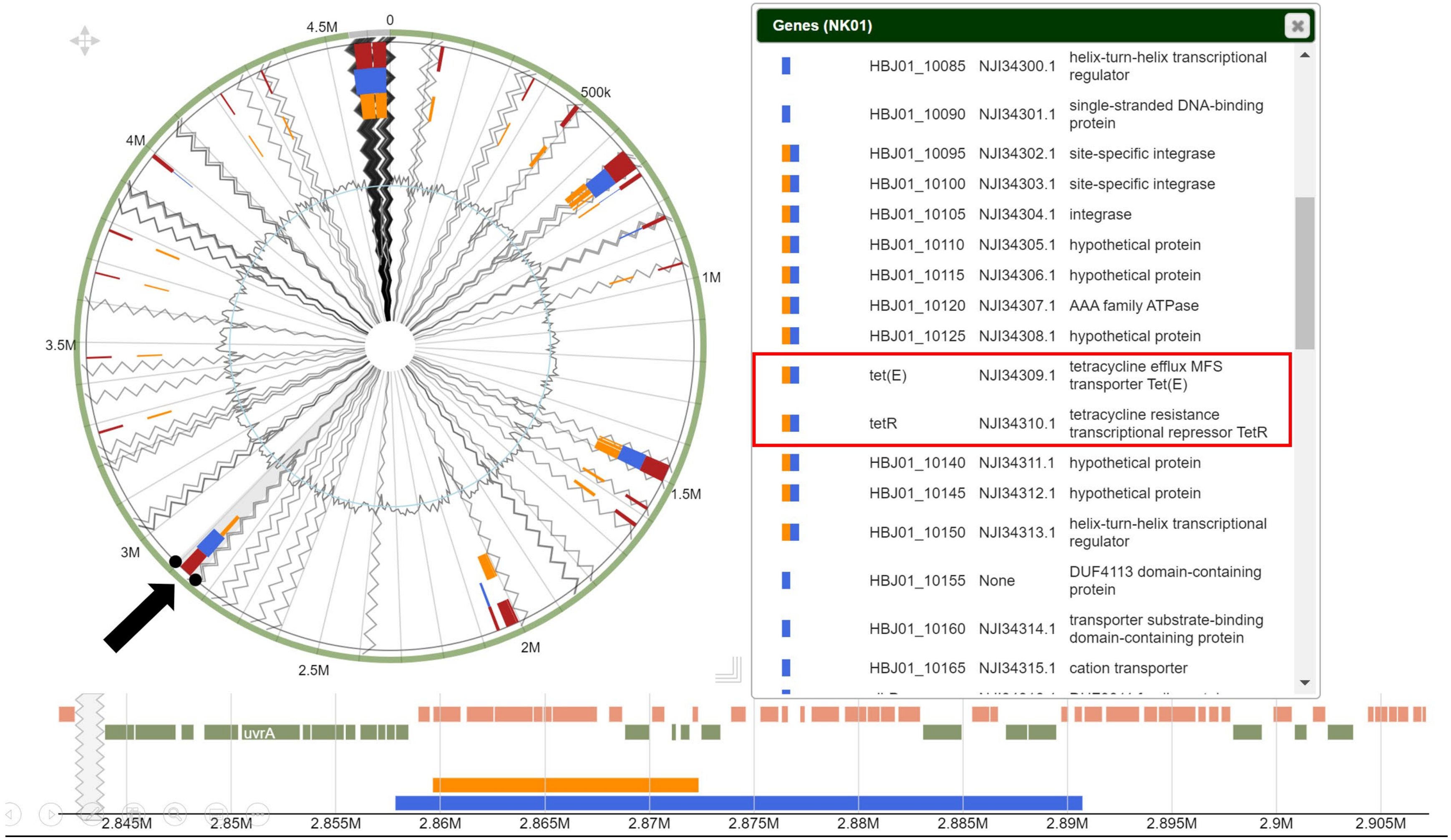

Figure S3 A 12-kb-long genomic island (GI) carrying antimicrobial resistance genes presented in the genome of *Aeromonas veronii* NK01. The figure was captured from IslandViewer4 output page. Red box showed tetracycline resistance genes (*tet*(E) and *tet*R) resided within this GI (depicted by arrow). The identical GI was also found in the genome of *A. veronii* UDRT09.
